# Abnormal mTOR Activity in Pediatric Autoimmune Neuropsychiatric and MIA-Associated Autism Spectrum Disorders

**DOI:** 10.3390/ijms23020967

**Published:** 2022-01-16

**Authors:** Ekaterina A. Trifonova, Zakhar S. Mustafin, Sergey A. Lashin, Alex V. Kochetov

**Affiliations:** 1 Federal Research Center Institute of Cytology and Genetics, Siberian Branch of the Russian Academy of Sciences, 630090 Novosibirsk, Russia; MustafinZS@bionet.nsc.ru (Z.S.M.); lashin@bionet.nsc.ru (S.A.L.); ak@bionet.nsc.ru (A.V.K.); 2 Natural Science Faculty, Novosibirsk National Research State University, 630090 Novosibirsk, Russia

**Keywords:** genetics, bioinformatics, autism spectrum disorder (ASD), autoimmune disorders (AIDs), mTOR signaling pathway, maternal autoantibody-related (MAR) ASD, maternal immune activation (MIA), pediatric autoimmune neuropsychiatric disorder associated with streptococcal infections (PANDAS)

## Abstract

Autism spectrum disorder (ASD) is a neurodevelopmental condition characterized by the early onset of communication and behavioral problems. ASD is highly heritable; however, environmental factors also play a considerable role in this disorder. A significant part of both syndromic and idiopathic autism cases could be attributed to disorders caused by mammalian target of rapamycin (mTOR)-dependent translation deregulation. This narrative review analyzes both bioinformatic and experimental evidence that connects mTOR signaling to the maternal autoantibody-related (MAR) autism spectrum and autoimmune neuropsychiatric disorders simultaneously. In addition, we reconstruct a network presenting the interactions between the mTOR signaling and eight MAR ASD genes coding for ASD-specific maternal autoantibody target proteins. The research discussed in this review demonstrates novel perspectives and validates the need for a subtyping of ASD on the grounds of pathogenic mechanisms. The utter necessity of designing ELISA-based test panels to identify all antibodies related to autism-like behavior is also considered.

## 1. Introduction

Autism spectrum disorder (ASD) is a neurodevelopmental condition characterized by the early onset of communication problems, including verbal communication, stereotypical behavior, and restricted interests. According to data from the Centers for Disease Control and Prevention (CDC), ASD affects approximately 1 in 54 children in the USA [1]. ASD is highly heritable, meaning that it has a strong genetic component and genetic factors play a consistently larger role than environmental factors [2]. At the same time, ASD genetics is so complex and heterogeneous that it has become a challenge for modern molecular genetics and bioinformatics. More than one thousand genes were identified and cataloged in the SFARI (Simon’s Foundation Autism Research Initiative) Gene database, which scored and ranked genes into one of four categories [3].

The syndromic form of ASD includes syndromes caused by monogenic mutations that significantly increase the risk of developing autism, but each of them has additional features not associated with ASD. It is now generally accepted that genetic causes can explain up to 25% of ASD cases; in the case when it is not possible to find genetic or other known mechanisms, the term “idiopathic autism” is used [4]. The significant genetic heterogeneity in ASD has sparked interest in identifying common signaling pathways and molecular mechanisms responsible for the disorder. With the help of animal models, it has come to be understood that disturbances in the structure, function, or formation of interneuronal connections—synapses—often occur in ASD. It turned out that some autism predisposition genes encode structural proteins of the synapse, such as Shank3 or neuroligins, while others encode proteins that regulate translation initiation. Fragile X syndrome and tuberous sclerosis are the most well-known syndromes associated with dysregulation of local translation in synapses [5]. One of the genes causing such a little-known syndrome is the mechanical target of rapamycin (mTOR), serine/threonine kinase, a central component of two multiprotein complexes, mTORC1 and mTORC2, which differ in protein composition and substrates. It has been shown that deregulation of the mTOR signaling pathway plays a role in the pathogenesis of many neurological disorders, such as epilepsy, autism, mental retardation, Alzheimer’s disease, and brain tumors [6].

Given the common neuropsychiatric manifestations of syndromic and idiopathic autism, it has been suggested that mTORC1 deregulation is a unified pathological mechanism of the disorder [7]. Indeed, higher activity of mTOR, ERK, and p70S6 kinase and lower activity of GSK3 and tuberin (TSC2) were observed in children with non-syndromic autism, suggesting an increase in Akt/mTOR activity in idiopathic ASD [8]. A decrease in interferon-, EGF-, and PDGF-production with simultaneous activation of the PI3K/AKT/mTOR and RAS/MAPK signaling pathways and translation in non-syndromic ASD was also demonstrated [9]. Therefore, a significant portion of both syndromic and idiopathic autism cases can be attributed to disorders caused by mTOR-dependent translation deregulation. The generally accepted behavioral diagnosis of ASD does not provide a practical possibility of subtyping pathological processes leading to the disorder, and genetic screening divides the spectrum into very small categories (usually less than 1%), without offering general mechanism-based therapy. This review analyzes autoimmune disorders leading to autism-like behavior, their connections with abnormal mTOR activity, and approaches to their diagnosis for subsequent immunomodulatory therapy.

## 2. Maternal Immune Activation (MIA)-Associated ASD

The teratogenic effects of maternal infections, such as rubella, cytomegalovirus, or Toxoplasma gondii, on the central nervous system are well established (reviewed [10]). Based on the meta-analysis, infections during pregnancy increased the risk of ASD by an average of 12%, especially in the case of severe infections that led to hospitalization. In addition, the risk of ASD was influenced by: (a) the type of infectious agent, (b) exposure time, and (c) the site of infection. Although only a relatively small percentage association between maternal immune activation (MIA) and ASD was found, the population influence of this association should be significant because of the high prevalence of infection during pregnancy worldwide. Moreover, it has been shown that for ASD children with intellectual disability, the correlation with MIA is even higher, which suggests a particularly strong effect of maternal infections in more severe cases of the disorder [11].

MIA can be induced experimentally using immunogens, such as polyinosine-polycytidic acid (poly (I: C)) and lipopolysaccharide (LPS). Poly (I: C) mimics viral infections by signaling toll-like receptor 3 (TLR3), which induces the production of type I interferons (IFN-α and IFN-β). LPS, in turn, mimics bacterial infections through TLR4 signaling, which stimulates subsequent production and secretion of TNF-α by macrophages [12]. Both poly (I: C) and LPS affect maternal cytokine signaling (e.g., interleukin-6), which, through the placenta, affects fetal brain development [13], blocking key pathways that prevent MIA-induced neurological and behavioral anomalies in ASD model systems [14]. As a result of MIA, the following characteristics of ASD appear in the developing brain: upregulation of cell cycle gene expression [13] and shortening of the cell cycle [15], increased cortical thickness [16] and brain size [17], altered expression of genes involved in neuronal migration [13], microglial abnormalities and enhanced microglial priming [17,18], impaired GABAergic signaling [19], and defects in prefrontal dendritic morphology [20].

The hypothesis that MIA disrupts fetal brain transcriptome regulation, leading to the same changes that are observed in children and adults with ASD, was tested in animal models [21]. It has been shown that MIA-deregulated genes in model mice are also deregulated in the cortex transcriptomes of children and adults with ASD. MIA downregulates the expression of ASD-associated genes, with the largest enrichments in genes bearing rare highly penetrant mutations. MIA also downregulates many of the genes that are constantly low expressed in the cerebral cortex of patients with ASD, which are canonically known for their role in late prenatal synapse development. Transcriptional and translational programs that are downstream targets of highly ASD-penetrant FMR1 and CHD8 genes are also strongly altered by MIA. MIA significantly upregulates the expression of a large number of genes involved in translation initiation, the cell cycle, DNA damage repair, and proteolysis processes that affect several key neurodevelopmental functions. Upregulation of translation initiation is a common feature of the autistic cortical transcriptome and has a direct impact on cell cycle processes. The key translation initiation gene, EIF4E, is the most MIA-deregulated of all ASD predisposition genes. This disturbance of translation initiation via alteration of the mTOR–Eif4e axis was additionally proved across MIA rodent models. MIA may increase risk of ASD by deregulating fetal brain gene expressions that are highly relevant to the pathogenesis of ASD [21].

The other highly ASD-penetrant heterozygous mutations in the *TSC1* or *TSC2* genes, key regulators of mTOR signaling, cause tuberous sclerosis (*TSC*). Of children affected by *TSC*, 40–50% develop ASD, and one possible explanation for this partial penetrance is the interaction between *TSC2* gene mutations and environmental risk factors. It has been shown that MIA and the mutant *TSC2* allele interact, disrupting fetal survival and social behavior in adult mice. In the experiments, the authors used *TSC2* +/−/Poly (I: C) model mice to simulate seasonal viral infections, and statistical analysis of the human *TSC* population revealed an association between high seasonal influenza activity in late pregnancy and the onset of autism in the child in addition to *TSC*. Altogether, the studies raise the possibility of a gene–environment interaction between heterozygous *TSC* gene mutations and MIA in the pathogenesis of tuberous sclerosis-related ASD [22].

The maternal fetal brain-reactive antibodies have been proposed as one possible mechanism of how MIA provokes the development of the ASD phenotype. Western blot hybridization of blood serum samples from mothers of ASD children, mothers of typically developing children, and mothers of children with developmental pathologies not related to ASD showed that the serum of ASD children’s mothers unambiguously binds to proteins of 37 kDa and 73 kDa in the fetal brain, especially in children with a regressive form of autism [23]. Subsequent studies have shown a correlation between maternal antibodies reactive to 39 kDa and 73 kDa proteins and the diagnosis of ASD, underdevelopment of expressive speech, and increased irritability in children [24].

Animal models have confirmed the role of maternal autoantibodies in the pathogenesis of ASD. Injecting serum from a mother of an ASD child to pregnant mice resulted in offspring with altered cognitive abilities and motor coordination [25]. Animal neuroanatomical studies have shown that prenatal exposure to autism-specific maternal autoantibodies increased stem cell proliferation in the subventricular zone (SVZ) of the embryonic neocortex, increased adult brain size and weight, and increased the size of adult cortical neurons [26].

It was demonstrated that the social behavior deficits in a mouse model of MIA could be temporarily rescued by the inflammatory response elicited by the administration of lipopolysaccharide (LPS) [27]. By contrast, an LPS-induced rescue of social deficits did not occur in the monogenic mouse models. The differences in responsiveness to the LPS treatment between the MIA and the monogenic models emerge from differences in the levels of cytokine production. LPS treatment in monogenic mutant mice did not induce amounts of interleukin-17a (IL-17a) comparable to those induced in MIA offspring; direct delivery of IL-17a into the somatosensory cortex dysgranular zone was sufficient to promote sociability in monogenic mutant mice as well as in MIA offspring. The data support a neuroimmune mechanism that underlies neurodevelopmental disorders in which the production of IL-17a during inflammation can ameliorate the expression of social behavior deficits by directly affecting neuronal activity in the central nervous system [27].

**Figure 1:**
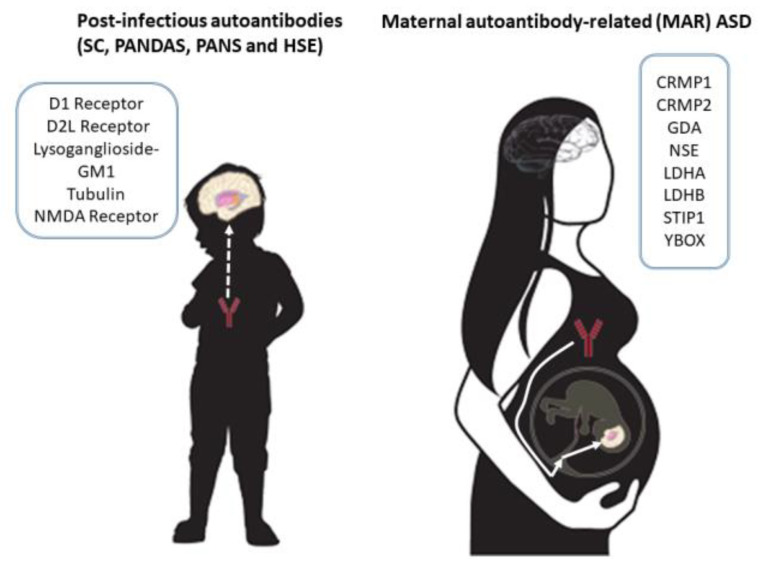
Autoantibody targets in post-infectious autoimmune neuropsychiatric disorders and MAR ASD. D1R, dopamine D1 receptor; D2LR, dopamine D2L receptor; neuronal surface antigen lysoganglioside-GM1; tubulin; NMDA receptor, N-methyl-D-aspartate receptor; CRMP1/CRMP2, collapsing response mediator proteins 1 and 2; GDA, guanine deaminase, cytosolic PSD-95 interactor; NSE, neuronspecific enolase; LDHA/LDHB, lactate dehydrogenase A and B; STIP1, stress-induced phosphoprotein 1; and YBOX, Y-box binding protein 1.

A recent study aimed to create a validated serological assay to identify ASD-specific maternal autoantibody patterns of reactivity against eight previously identified proteins (CRMP1, CRMP2, GDA, NSE, LDHA, LDHB, STIP1, and YBOX) that are highly expressed in the developing brain [28} (Figure 1). The authors developed an ELISA test for each of the protein antigens and determined patterns of reactivity a highly significant association with ASD. Three main patterns associated with maternal autoantibody-related (MAR) ASD were discovered: CRMP1 + GDA (ASD% = 4.2 vs. TD% = 0, OR 31.04, *p* =< 0.0001), CRMP1 + CRMP2 (ASD% = 3.6 vs. TD% = 0, OR 26.08, *p* = 0.0005) and NSE + STIP1 (ASD% = 3.1 vs. TD% = 0, OR 22.82, *p* = 0.0001). Additionally, it was found that maternal autoantibody reactivity to CRMP1 significantly increases the odds of a child having a higher Autism Diagnostic Observation Schedule (ADOS) severity score (OR 2.3; 95% CI: 1.358–3.987, *p* = 0.0021). This was the first work that used machine learning subgroup discovery to identify MAR ASD-specific patterns with 100% accuracy for up to 18% of ASD cases in this study participants [28].

In general, it has been shown that specific maternal autoantibodies directed against the fetal brain is associated with ASD, and exposure to these autoantibodies during pregnancy leads to neuroanatomical changes and the behavioral phenotype of ASD in rodent models. Thus, the influence of maternal autoantibodies is probably a decisive factor for part (up to 18%) of the autism spectrum [28,29]; the cap-dependent translation initiation gene, *EIF4E*, is one of the most MIA-dysregulated of all ASD-associated genes, and the Tsc2/mTOR/eIF4e pathway is the most MIA-upregulated pathway [21].

## 3. Post-Infectious Autoimmune Neuropsychiatric Disorders: Sydenham’s Chorea, PANDAS, PANS, and Herpes Simplex Encephalitis

Infectious inflammation and post-infectious autoimmune sequelae have increasingly been considered as pathogenic mechanisms for neuropsychiatric disorders. Sydenham’s chorea (SC), formerly known as St. Vitus Dance, post-streptococcal autoimmune sequela, represents a model for this pathogenic condition. In SC, autoimmune sequelae of a streptococcal infection result in neuroinflammation, particularly the basal ganglia nuclei. Phenotypically, this dysfunction manifests itself as a constellation of involuntary movements and psychiatric symptoms that may be alleviated by immunomodulatory therapies. PANDAS (Pediatric Autoimmune Neuropsychiatric Disorder Associated with Streptococcal infections) has been suggested as a subtype of SC with the same pathogenic mechanism and a unique set of prevalently psychiatric symptoms [30]. It is worth noting that streptococci are not unique in their ability to provoke autoimmune neuropsychiatric sequela; among alternative inducers are influenza and chickenpox viruses and mycoplasma. The whole spectrum of such syndromes is called PANS (pediatric acute-onset neuropsychiatric syndrome) [30].

Neuroimmune diseases in childhood are particularly difficult to identify. “PANS and PANDAS are sometimes misdiagnosed as OCD, Tourette’s syndrome, autism spectrum disorder (ASD), ADHD, anorexia, and other psychiatric disorders. The presence of obsessive rituals and interests, rigidity around routines, and impaired eye contact may be confused with ASD in young children,” said Casoli Reardon, MD [31]. “PANDAS may be more difficult to recognize in a child with autism—due to overlapping symptoms. Or it may be mistaken for classic OCD, which commonly co-occurs with autism,” said Susan Swedo, MD, who coined the term PANDAS in 1998 [32]. The case of a six-year-old boy who remained severely disabled for five years of his life due to misdiagnoses of pediatric acute-onset neuropsychiatric syndrome as ASD was reported in 2018 [33].

Therefore, when symptom onset is abrupt, PANDAS and PANS are two disorders that should be considered primarily as diagnoses. The diagnostic criteria for PANS is defined as an abrupt onset of OCD or severely restricted food intake and the presence of at least two of the following seven categories: (1) anxiety; (2) emotional lability and/or depression; (3) irritability, aggression, and/or severely oppositional behaviors; (4) behavioral (developmental) regression; (5) deterioration in school performance (related to attention deficit hyperactivity disorder-like symptoms, memory deficits, cognitive changes); (6) sensory or motor abnormalities; (7) somatic signs and symptoms, including sleep disturbances, enuresis, or increased urinary frequency [34]. Since SC has a well-established pathogenic mechanism connecting streptococcal infections with autoimmune-induced neuropsychiatric symptoms, it has been used as a biological model to better understand PANDAS and PANS. Targets shown to overlap between Sydenham chorea and PANDAS were used to develop the Cunningham Panel, a set of blood tests utilized for measuring immune dysfunction, related to neuropsychiatric conditions associated with an infectious trigger [35,36].

The Cunningham Panel includes five assays performed on a serum sample from blood collected in glass tubes. Four assays measure human serum immunoglobulin G (IgG) levels by enzyme-linked immunosorbent assays (ELISA) directed against (1) dopamine D1 receptor (D1R), (2) dopamine D2L receptor (D2LR), (3) lysoganglioside-GM1, and (4) tubulin. A fifth assay is a cell stimulation assay which measures the ability of a patient’s serum immunoglobulin G (IgG) to stimulate calcium/calmodulin-dependent protein kinase II (CaMKII) activity in human neuronal cells [37] (Figure 1).

Herpes simplex virus 1 (HSV1) encephalitis (HSE) is an uncommon serious condition in adults and children. Complications after HSE can be severe and include chorea or epileptic encephalopathy [38]. It has been shown that some patients with HSE have immunoglobulin G (IgG) autoantibodies against the N-methyl D-aspartate receptor (NMDAR), although their clinical phenotype did not differ from antibody-negative patients. NMDAR antibodies have been used to define an autoimmune encephalitis characterized by seizures, encephalopathy, psychosis, dyskinesia, and autonomic dysfunction [39] (Figure 1).

Fast and significant loss of social and communication skills is typical for regressive autism, especially when it occurs before the age of three. At the same time, regressive ASD, childhood disintegrative disorder, early onset schizophrenia, and all stages of anti-NMDA-receptor encephalitis share core symptoms. [40,41]. Creten and colleagues [40] suggested that patients previously diagnosed with early onset schizophrenia or regressive ASD might need to be re-examined for anti-NMDA-receptor encephalitis because, unlike autism, early diagnosis and treatment of NMDAR-Ab encephalitis is associated with a much better outcome [42].

mTOR signaling connects bioenergetic and biosynthetic metabolism to immune responses. Initially, the main role of mTOR was to regulate cellular metabolism; as immune systems developed, the immune regulating signaling pathways also explored the mTOR pathway as a means to detect and control pathogen replication, and pathogens have targeted this pathway as a means to improve their reproduction (reviewed [43]). It is known that all viruses require host ribosomes to translate their mRNAs. Viruses producing methyl-7 (m⁷) GTP-capped mRNAs, like HSV-1, stimulate cap-dependent translation by activating mTORC1 to inhibit the translational repressor 4E-BP1. Moreover, HSV-1 encodes an Akt surrogate kinase Us3 with overlapping substrate specificity to activate mTORC1, stimulating translation and viral replication [44]. Streptococcus infections are a frequent complication of viral infections and as a result occur against the background of hyperactivated mTOR signaling and compromised autophagy. It was demonstrated that inhibition of the PI3K/AKT/mTOR signaling pathway or overexpressed Beclin1 alleviates reinfection of *S. pneumonia* after influenza A virus infection [45]. Group A Streptococcus (GAS), one of the most common pathogens and PANDAS inductor, utilizes internalization into cells as a major immune evasion strategy. mTOR-dependent autophagy is an important strategy for non-phagocytes to eliminate intracellular bacteria, so GAS multiplication is directly related to mTOR signaling activity [46,47]. Moreover, during GAS-host interaction, GAS regulates multiple autophagy pathways, including mTOR, using distinct regulators, such as streptolysin O and Nga, to survive in host cells [48].

It was proposed that strategically targeting mTOR could influence the immune-mediated clinical cerebral outcomes of host–protozoan interactions and additionally act to limit protozoan replication [49]. Treatment with mTOR inhibitor rapamycin increased survival, blocked breakdown of the blood–brain barrier and brain hemorrhaging, decreased the influx of both CD4(+) and CD8(+) T cells into the brain and the accumulation of parasitized red blood cells in a mouse model of cerebral malaria (CM), experimental CM [50]. Given the involvement of mTOR signaling in both viral and bacterial infection process, a similar strategy for preventing cerebral complications of the other infections might also be very productive.

## 4. mTOR Signaling as a Key Player in Immune, Microbiome, and Behavior Abnormalities in ASD

Neuroinflammation or immune dysregulation, microglial activation, genetically linked neurotransmission, mitochondrial dysfunctions, and the mTOR signaling pathway were designated as the primary targets for treating and preventing ASD [51]. However, in fact, mTOR signaling pathway gene mutations and/or mTOR dysregulation might be responsible for immune disturbance, microglial activation and mitochondrial dysfunctions at the same time.

As a hub integrating multiple intracellular, plasma membrane-associated, and extracellular signals regulating T cell viability, mTOR is recognized to be related to T cell responses. CD4+ T helper (Th) cells act as an important part of the adaptive immune system, naıve CD4+ T cells respond to different antigen stimulation by differentiating into distinct effector cell subsets, including Th1,Th2, Th17, and follicular Th cells (Tfh), or into regulatory T cells (Tregs), which prevent excessive immune reactions [52]. In the complete absence of mTOR, naıve T cells showed normal activation markers upon T cell receptor (TCR) stimulation, but failed to differentiate into Th1, Th2, and Th17 cells. This was associated with decreased phosphorylation of lineage-selective STAT proteins and insufficient induction of lineage-selective master transcription factors [53]. Further studies showed that abrogating mTORC1 activity in naive T cells by genetic deletion of Rheb, an upstream activator of mTORC1, would block the differentiation process of Th1 and Th17 cells, while Th2 differentiation would not be affected. Rheb-deleted T cells express increased levels of suppressor of cytokine signaling 3 (SOCS3), a negative regulator of STAT signaling, and silence of SOCS3 expression can restore Th1 differentiation [53].

On the other hand, it was reported that treating T cells with mTOR inhibitor rapamycin during expansion in the absence of exogenous TFG-β also resulted in a relatively high percentage of Tregs in the culture system. Both the de novo generation of Foxp3+ cells from naıve T cells and the selective expansion of regulatory cells from effector cells might contribute to the effect of rapamycin on Treg enrichment. It was proposed that rapamycin could enhance the selective pressure for Treg expansion [54]. Overall, contemporary research reviewed [52] indicates that mTOR signaling activation is a positive regulator for the differentiation of CD4+ effector T cells and a negative regulator for Treg differentiation, and dysregulated mTOR signaling is involved in a number of autoimmune diseases.

Microglial activation is a prominent feature of neuroinflammation, which is present in both neurodegenerative diseases and ASD. In a recent study, the mechanism by which lipopolysaccharide (LPS) affects microglial autophagy and the effects of autophagy on the production of pro-inflammatory factors in microglial cells were examined. The authors found that autophagic flux was suppressed in LPS-stimulated N9 microglial cells, and LPS significantly decreased Vps34 expression in N9 microglial cells by activating the PI3KI/AKT/mTOR pathway without affecting the levels of lysosome-associated proteins and enzymes. Furthermore, LPS-induced neuroinflammation was significantly ameliorated by treatment with the autophagy inducer (and mTOR inhibitor) rapamycin both in vitro and in vivo [55].

Mitochondria are major substrates of autophagy; however, their connection with mTOR remains insufficiently clear. Using TSC as a genetic model, the impact of aberrant mTOR signaling on mitochondrial dynamics, function, and turnover in neurons in vitro and in vivo was investigated [56]. The authors found deficits in all domains of respiratory function and a significant decrease in the mitochondrial membrane potential, particularly in mitochondria that localized to the dendritic and axonal compartments; moreover, the turnover of mitochondria through autophagy, or mitophagy, was critically impaired both in the axon and globally. Indeed, the treatment of Tsc2-deficient neurons and mice with the mTOR inhibitor rapamycin restores the mitochondrial phenotypes, including mitochondrial mass, transport, and mitophagy [57]. It was also shown that rapamycin ameliorates defects in mitochondrial fission and mitophagy in glioblastoma cells [56] and alleviates cognitive impairment in murine vascular dementia through the enhancement of mitophagy by the PI3K/AKT/mTOR axis [58].

The gut microbiota has also been suggested to play an important role in ASD. Gut microbiota from human donors with ASD or typically developing controls were transplanted into germfree mice and it was revealed that colonization with ASD microbiome is sufficient to induce well-recognized autistic behaviors. It was proposed that the gut microbiota manages behaviors in mice by the production of neuroactive metabolites [59]. Enteric short-chain fatty acids are a basic type of bacterial signaling molecules produced from fermentation of carbohydrates, odd-chain fatty acids, and proteins. The most prominent of these are acetic acid (AA), butyric acid (BA), and propionic acid (PPA) [60]. Besides being a secondary metabolite of gut microbiota, PPA is widely used as a food preservative. The effect of PPA was investigated on human neural stem cells proliferated to neuropsheres. The neurosphere diameter was increased after PPA treatment to (193.47 um ± 6.673 um) versus (154.16 um ± 9.95 um) in control. Likewise, PPA receptor GPR41 and pro-survival p-Akt protein were increased while PTEN (Akt inhibitor) levels decreased to (0.42 ug/ul ± 0.04 ug/ul) at 2 mM PPA compared to (0.83 ug/ul ± 0.09 ug/ul) in control. In the whole, PPA induced gliosis and neuroinflammation through upregulation of the PTEN/AKT/mTOR pathway [61].

On the other hand, butyric acid (BA), which modulates mitochondrial function, was proposed as a neuroprotective agent. On the lymphoblastoid cell line (LCL) model of ASD (AD-A) and another subset of control LCLs (AD-N), it was demonstrated that BA significantly increased its respiratory parameters linked to ATP production in AD-A LCLs but not in AD-N LCLs. The high levels of BA (1 mM) enhanced the expression of genes involved in mitochondrial fission (PINK1, DRP1, FIS1) and physiological stress, including mTOR. These data suggest that microbiome-derived BA can enhance mitochondrial function under physiological stress conditions or mitochondrial dysfunction and modulate the mTOR pathway [62].

It is worth noting that, unlike immune dysregulation, microglial activation, and mitochondrial dysfunctions associated with autism, microbiota dysbiosis is not a consequence but, perhaps, the cause of mTOR signaling pathway activation. It was even hypothesized that ASDs are induced by the early life alterations in intestinal microbiota in genetically predisposed sub-populations, which may have significant implications in ASD root, diagnosis, prophylaxis, and treatment [63].

ASD is commonly recognized as highly heterogeneous with respect to phenotypes, genetics, and etiology. Dysregulation of the mTORC1 pathway has been identified in numerous ASD syndromes, such as fragile X syndrome, tuberous sclerosis, PTEN hamartoma tumor syndrome, a set of syndromes named RASopathy (including type 1 neurofibromatosis and other mutations in the RAS/MAPK pathway genes, with a very similar manifestation), Angelman syndrome, Rett syndrome, and Phelan–McDermid syndrome [5]. Given the similarities in neuropsychiatric symptoms between syndromic and idiopathic forms of ASD, mTORC1 dysregulation has been hypothesized to be a common pathological mechanism. Our gene-set and gene network analyses revealed that on the whole, 222 out of 281 (79%) high-scored genes from the SFARI (Simon’s Foundation Autism Research Initiative) Gene database [3] were connected with mTOR signaling activity and/or dependent on vitamin D3 availability directly or indirectly [64]. Vitamin D3 hormone has been associated with autism based primarily on a correlation between autism incidences and low levels of vitamin D3 in populations. However, the effect of vitamin D3 on ASD symptoms may be also connected with 1,25-dihydroxyvitamin D ability to stimulate expression of DNA damage-inducible transcript 4 (DDIT4), which is a potent mTOR suppressor [65]. At the same time, a literature review demonstrated an inverse association between vitamin D and the development of several autoimmune diseases (AID), such as systemic lupus erythematosus, thyrotoxicosis, type 1 diabetes, multiple sclerosis, iridocyclitis, Crohn’s disease, ulcerative colitis, psoriasis vulgaris, seropositive rheumatoid arthritis, and polymyalgia rheumatic [66]. The autoimmune and chronic inflammatory diseases are complex conditions with an individual genetic predisposition providing a rate of heritability close to that of ASD. We have bioinformatically demonstrated that autism spectrum and autoimmune disorders do share predisposition gene signatures due to mTOR signaling pathway controlling expression. Our network analysis revealed that the great majority (up to 67%) of AID genes are related to the mTOR signaling pathway directly or indirectly [67]. Our bioinformatic analysis and reviewed published data allow us to hypothesize that both a certain part of ASD and AID comprise a connected set of disorders sharing a common aberrant pathway (mTOR signaling) rather than a vast set of different disorders. Furthermore, a certain part of the autism spectrum might be a specific type of autoimmune disorder with an early manifestation of a unique set of predominantly behavioral symptoms [67].

**Figure 2:**
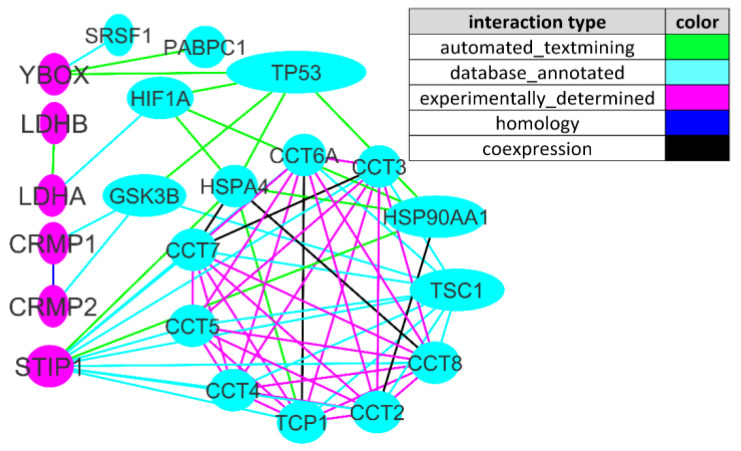
mTOR signaling pathway genes interacting with MAR ASD genes. The edges of the network are colored according to the type of interaction that had the highest confidence score. There were five dominant types of interactions: automated textmining (colored green), annotated database (colored cyan), experimentally determined (colored pink), homology (colored blue), and coexpression (colored black). mTOR signaling genes colored cyan, MAR ASD genes colored pink.

We analyzed the interactions between the mTOR signaling pathway and MAR ASD genes coding for ASD-specific maternal autoantibody target proteins. The initial set of 266 genes (258 mTOR signaling pathway genes [68] and 8 MAR ASD genes [28]) was analyzed by STRING tools. By means of Cytoscape, the edges of the network with the lowest confidence score and genes without intergroup interactions were gradually removed. For the 0.9 (highest confidence) cutoff, the network contained 22 genes and 67 interactions (i.e., network edges) between them. (Figure 2).

We found that six (*CRMP1*, *CRMP2*, *LDHA*, *LDHB*, *STIP1*, and *YBOX*) out of eight MAR ASD genes are tightly interwoven with the mTOR pathway, and three (*LDHA*, *LDHB*, and *STIP1*) out of eight are directly mTOR-modulated [69]. STIP1 (stress-induced phosphoprotein 1), a co-chaperone of Hsp70 and Hsp90 (heat shock proteins), together with CRMP2, were among the most upregulated proteins during glutamate-induced neurogenesis [70]. LDHA/LDAB (lactate dehydrogenase A and B) is an oxidoreductase converting pyruvate to lactate that leads to rapid energy production. Notably, increased levels of pyruvate and lactate are often suggested as a marker for mitochondrial dysfunction given that neurons are strongly energy-dependent and therefore susceptible to mitochondrial deficiencies [71]. YBOX (Y-box-binding protein 1) participates in DNA and RNA-binding during mRNA processing, splicing, aand transcription and transcription regulation and directly interacts with methyl-CpG-binding protein 2 (MeCP2), which is the cause of majority of Rett syndrome cases [72]. CRMP1 and CRMP2 (collapsin response mediator proteins 1 and 2) are two out of five phosphoproteins that are involved in the cytoskeleton, microtubuli, and actin filament organization [73]. The most connected MAR ASD gene of the network with a node degree of 11 was STIP1 (Figure 2). It directly interacts with TSC1 (tuberous sclerosis 1), also known as hamartin, which is an important upstream mTOR inhibitor [5], and CCT2 (chaperonin containing TCP-1), CCT3, CCT4, CCT5, CCT6A, CCT7, and CCT8, which constitute a large multi-subunit complex mediating protein folding in eukaryotic cells [74] (Figure 2). It is worth mentioning that at least one mTOR-connected gene presented in each of the three main patterns associated with MAR ASD (CRMP1 + GDA, CRMP1 + CRMP2, and NSE + STIP1) [28}, namely CRMP1, CRMP2, and STIP1.

## 5. Challenges, Limitations and Future Prospects

For several decades, autism spectrum disorders have been diagnosed and classified based on behavioral criteria. Recent bioinformatic and molecular biological investigations opened up novel perspectives and validated the need for a subtyping of the autism spectrum and other pediatric neuropsychiatric disorders on the grounds of pathogenic mechanisms. In this review we partially answered the question of which pathways mediate the interplay between extent of inflammation and cognitive/behavior impairments that was raised in [75]. Undoubtedly, one of the most important pathways connecting the immune and the central nervous systems is the mTOR signaling pathway. Further attempts to modulate this huge and branched pathway at different stages with natural and pharmacological inhibitors may become one of the promising research directions in the future in order to address abnormal mTOR activation therapeutically.

Unlike immune dysregulation and behavior impairments associated with ASD, dysbiosis of the gut microbiota is not a consequence but, perhaps, the cause of mTOR pathway activation. The objective limitation of this direction of research development is the lack of non-invasive analysis of the small intestine parietal microbiome. The elaboration of the methods for assessing the intestinal microbiome and bioinformatic analysis might lead to the creation of a comprehensive probiotic therapy of the disorders caused by abnormal mTOR activity in the future.

Traditionally, genetic screening for mutations in the autism predisposition genes comes first in the diagnostic route for patients with autistic behavior. Without disputing the importance of identifying highly penetrant mutations in ASD genes, we believe that the next step in the diagnostic route should be screening for blood, CSF and magnetic resonance imaging markers of inflammation, in particular, specific autism-like behavior associated anti-neuronal antibodies. In fact, by analogy with already available panels for analyzing mutations in the autism predisposition genes, ELISA-based test panels should be designed to identify all autism-like behavior-related antibodies. If there are any signs of active inflammation or ASD-related antibodies, immunomodulatory treatment should be considered.
